# Reduction en-masse of inguinal hernia with strangulated obstruction

**DOI:** 10.2349/biij.5.4.e14

**Published:** 2009-10-01

**Authors:** H Ravikumar, S Babu, MJ Govindrajan, A Kalyanpur

**Affiliations:** Teleradiology Solutions, Bangalore, India

**Keywords:** Inguinal hernia, hernial sac, reduction en masse, properitoneal space, closed-loop obstruction

## Abstract

“Reduction en masse of inguinal hernia” means reduction/migration of a hernial sac into the properitoneal space. We report the CT findings in a case of reduction en masse with strangulated obstruction. CT scan demonstrated a hernial sac with fibrous constriction band at the neck, situated in the properitoneal space superior to the inguinal region, causing closed-loop obstruction. The hernial sac contained thickened bowel loop with wall enhancement and fluid suggestive of incarceration/strangulation. We propose to call this, ‘The properitoneal hernial sac sign’, defined as “Presence of a hernial sac in the properitoneal space (and not in the inguinal/femoral canal) containing an obstructed/incarcerated bowel loop and causing small bowel obstruction” to identify “reduction en masse of inguinal hernia”.

## INTRODUCTION

“Reduction en-masse of inguinal hernia”, means reduction/migration of a hernial sac along with the incarcerated bowel into the properitoneal space [1] and is likely produced by forcible attempts at reduction. Occasionally, it can also be spontaneous. There is usually a history of difficult reductions, the last one being especially difficult, after which the symptoms of intestinal obstruction occur. The hernia appears to have been reduced but the signs of bowel obstruction persist.

**Figure 1:**
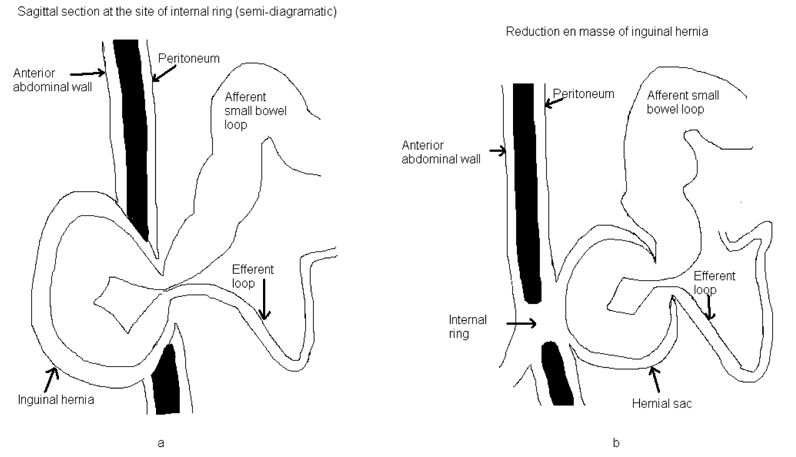
(a) Diagram of inguinal hernia at the site of inguinal ring. (b) Diagram of reduction en masse - A hernial sac is seen containing incarcerated bowel loop between the parietal peritoneum and anterior abdominal wall, the properitoneal sac.

A hernial sac containing incarcerated bowel loop is seen between the parietal peritoneum and anterior abdominal wall, the properitoneal sac (Figure 1).

## CASE REPORT

A 62 year-old male had a bulge in the left groin for the last two years. There was a history of repeated reductions. He presented with severe abdominal pain and vomiting after an episode of forcible reduction, for which a CT scan (5mm axial sections with intravenous contrast), was performed.

**Figure 2:**
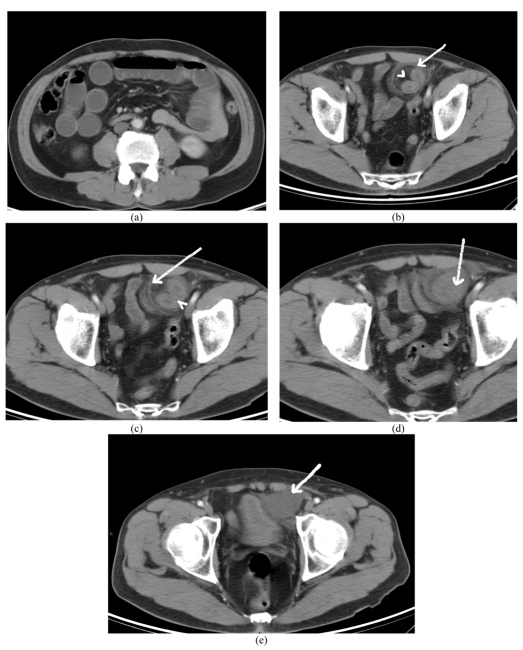
(a) CT scan of the abdomen demonstrating dilated small bowel loops suggestive of bowel obstruction. (b) The hernial sac [arrow] containing thickened bowel loop and mesenteric fat [arrowhead] is seen in the properitoneal space, superior to the left inguinal region. (c) A fibrous constriction band is seen around the neck of the hernial sac [arrow] .Note enhancement of bowel wall [arrowhead]. (d) The inferior aspect of the hernial sac containing bowel [arrow]. (e) Fluid in the hernial sac [arrow]. Wall enhancement of the bowel loop and fluid in the hernial sac suggest incarceration and strangulation.

The CT scan demonstrated a hernial sac in the properitoneal space, between the parietal peritoneum and anterior abdominal wall (Figure 2). The neck of the hernial sac showed a fibrous constriction band (Figure 2c) and there were features of closed loop small bowel obstruction. The hernial sac contained thickened bowel loop with wall enhancement and fluid suggestive of incarceration and strangulation (Figures 2c, 2d, and 2e). The patient was operated on and the hernial sac was found to contain ischemic small bowel loop, which was resected and end-to-end anastomosis was performed.

## DISCUSSION

Reduction en-masse of inguinal/femoral hernia can be defined as reduction of the hernial sac together with its intestinal contents so that the bowel still remains incarcerated [2]. It is a rare form of acute intestinal obstruction that few surgeons get to see and with which many radiologists are unfamiliar. It has been quoted by Pearse to occur in approximately 1 of 13,000 hernias [3].

Reduction en-masse of hernia is quite rare as a result of early repair of hernias and abandonment of forcible reduction [4]. Most unusual are the spontaneous reductions that have been documented.

Clinically, a tender mass can be palpated either high in the inguinal canal, above the inguinal ring or in the lower abdomen, on the side of reduction. Early surgical intervention is necessary as prognosis is not always good due to the delay in time from onset of symptoms and surgery, more so in spontaneous reductions.

Casten and Bodenheimer postulated that reduction en masse can occur only if there is a relatively unyielding neck of the sac and a lax internal ring [5]. Fibrosis is probably produced by recurrent trauma from difficult reductions. Pearse concluded that a preformed space between the parietal peritoneum and anterior abdominal wall, the properitoneal sac, or diverticulum was present in many cases, while Millard suggested that such a sac was equally likely to be produced by forcible attempts at reduction [6]. There is usually a history of difficult reductions, the last being more difficult, after which the symptoms of intestinal obstruction fail to subside or subside only temporarily [7, 8].

In the above case, the bowel, along with hernial sac, would appear to have been pushed back into the properitoneal space by repeated reductions. We propose to call this, ‘The properitoneal hernial sac sign’, defined as “Presence of a hernial sac in the properitoneal space (and not in the inguinal/femoral canal) containing an obstructed/incarcerated bowel loop and causing small bowel obstruction” to identify “Reduction en masse of herniae”. The properitoneal space may have been pre-formed by similar past incidents and later on, development of fibrosis at the neck of sac may have caused small bowel obstruction.

We were unable to provide details of the MDCT or intra-operative images as ours is a teleradiology service. Despite its numerous advantages, the expansion of telemedicine may pose an increasing challenge to collaboration, research and expansion of medical knowledge unless measures are put in place to overcome them.

In summary, reduction en-masse of inguinal hernia is a clinical diagnosis which can be confirmed by CT. Reduction en-masse of hernia should be considered as a cause of acute intestinal obstruction in patients with persistent bowel obstruction following reduction of inguinal/femoral hernias. The proposed ‘properitoneal hernial sac sign’ helps in identifying and confirming “reduction en-masse of hernia”, likely to be produced by forcible attempts at reduction. Rarely, it can also occur spontaneously, where the prognosis is bad.
